# Evidence of myogenic vasoconstriction in human bone vasculature

**DOI:** 10.14814/phy2.70278

**Published:** 2025-03-20

**Authors:** Adina E. Draghici, Matthew R. Ely, Jason W. Hamner

**Affiliations:** ^1^ Department of Physical Medicine and Rehabilitation Harvard Medical School Boston Massachusetts USA; ^2^ Cardiovascular Research Laboratory Spaulding Hospital Cambridge Cambridge Massachusetts USA; ^3^ Schoen Adams Research Institute at Spaulding Rehabilitation Boston Massachusetts USA

**Keywords:** bone blood flow, leg dependency, myogenic vasoconstriction, near infrared spectroscopy, tibial vasculature

## Abstract

Despite the critical importance of blood flow for bone, mechanisms regulating bone vasculature are poorly understood. Myogenic vasoconstriction is an important regulatory mechanism that is engaged in most daily activities, but our understanding primarily derives from animal work and/or other vascular beds. In young healthy adults, we employed two levels of leg dependency to engage myogenic vasoconstriction. We measured tibial blood content via near‐infrared spectroscopy (total hemoglobin, ΔtHb) and contrasted it to whole leg flow via popliteal blood flow velocity (LBV) via Doppler ultrasound. Myogenic vasoconstriction was engaged by lowering the leg below heart level (supine to upright to dependent), resulting in increased leg perfusion pressure as assessed by brachial mean pressure adjusted for the hydrostatic pressure from the heart to the tibia. Increased leg perfusion pressure in both positions (Δ30.1 ± 1.36 and Δ42.1 ± 1.16 mmHg; *p* < 0.01) was accompanied by graded declines in LBV (Δ‐1.88 ± 0.21 and Δ‐2.98 ± 0.27 cm/(s*beat); *p* < 0.01), indicating whole limb myogenic vasoconstriction. Tibial hemoglobin content did not change (ΔtHb: −0.28 ± 1.76 and 1.26 ± 2.33 μM; *p* > 0.5), indicating myogenic vasoconstriction was evident, but of lower magnitude compared to the whole leg. These results indicate that myogenic vasoconstriction plays an active role in regulating the tibial vasculature, but with a less robust response compared to the whole leg.

## 1 INTRODUCTION

The recognized importance of the bone vasculature dates to 1674 when Antoine van Leeuwenhoek, a founder of microscopic science, observed small veins on the surface of a cow shinbone (Leeuwenhoeck, 1674). However, over the next three centuries, the study of bone vasculature and its significance to bone health has been unusually slow, in part due to lack of adequate techniques. Nonetheless, it is now axiomatic that the extensive vascular network in bones is indispensable for adequate blood supply for nearly all skeletal functions (McCarthy, 2006). This broad understanding does not mean that we have complete knowledge of bone blood flow regulation. The very dense, compact nature of bone makes the study of bone circulation difficult; hence, almost the entirety of our knowledge derives from invasive animal work with very little in humans.

Animal data suggest that arteriolar smooth muscle in bone responds as expected to infused vasodilators and vasoconstrictors, with vasodilators increasing (Prisby et al., 2007) and vasoconstrictors decreasing (Azuma, 1964; Dean et al., 1992; Driessens & Vanhoutte, 1979; Shaw, 1963; Stein et al., 1958) blood flow. However, it remains unknown whether these pharmacologic responses reflect physiologic mechanisms. For example, we previously described vasoconstrictor responses to a physiologic sympatho‐excitatory stimulus in tibial bone in humans (Draghici & Taylor, 2021) that suggested differences in how the bone vasculature responds in comparison to the whole limb. An additional vasoconstrictor mechanism crucial for local blood flow regulation is myogenic constriction, which regulates tissue perfusion and protects downstream arterioles and capillaries from damage due to elevated perfusion pressure (Davis & Hill, 1999; Jackson, 2021; Johnson, 1986). Myogenic vasoconstriction is of particular importance since it is an autochthonous mechanism at the level of the smooth muscle that is engaged when vascular transmural pressure is increased and is demonstrated in most vascular beds (Kooijman et al., 2007; Okazaki et al., 2005; Snyder et al., 2012). A handful of studies have suggested myogenic responses in human tibial bone, such as myogenic vasodilatory responses to physiological stimuli (i.e., head down tilt and lower‐body negative pressure) that decrease transmural pressure (Becker et al., 2018; Siamwala et al., 2017). Our own recent work was suggestive of a compensatory role for myogenic vasoconstriction in regulating blood flow to the tibia in those with absent sympathetic control, that is, adults with spinal cord injury (Sukhoplyasova et al., 2024). However, there is no prior work that has directly characterized myogenic vasoconstriction in human bone.

Therefore, we examined myogenic vasoconstriction in the tibia in young healthy adults via changes in body position, that is, leg dependency. Responses in the tibia were compared to those in the whole leg, which are reflective predominantly of skeletal muscle responses. During two levels of increased perfusion pressure in response to leg dependency, we monitored beat‐to‐beat blood pressure, whole leg blood flow via Doppler ultrasound, and tibial blood content via our custom‐made near‐infrared spectroscopy (NIRS) system, as described in our prior work (Draghici et al., 2018). We hypothesized that changes in tibial blood content would be evidenced in response to leg dependency as in the whole leg, but with distinct characteristics based on our prior observations (Draghici et al., 2024; Draghici & Taylor, 2021).

## 2 METHODS

Sixteen young healthy individuals were enrolled in the study (8 M, 8 F; 27 ± 5 yrs.). All individuals had body mass index ≤29.9 kg/m^2^, were free of cardiovascular and neurological diseases, and had no tibial fractures in the past year. All volunteers had a complete medical history and were instructed to refrain from consuming caffeine and participating in exercise 24 h prior to testing.

### 2.1 Protocol and measurements

Subjects were instrumented with a standard 5‐lead ECG for continuous heart rate and oscillometric arm cuff for standard measures of brachial arterial pressure (Dinamap Dash 2000, GE). Popliteal artery blood flow velocity (i.e., representing whole leg blood flow to muscle and bone) was measured via a 4‐MHz Doppler probe (Doppler BoxX, DWL) at the popliteal fossa of the right leg. Even though changes in vessel diameter would confound the estimate of vessel response, previous work has shown that diameters remain constant during postural changes (Schlager et al., 2011), and thus the changes observed in total whole leg blood flow velocity should reflect changes in whole leg blood flow. All signals were digitized at 1 kHz and stored for subsequent offline analysis (ADInstruments, PowerLab). Tibial blood content was assessed using the custom‐made NIRS system placed mid shaft (50% of tibial length), on the anteromedial side of the tibia, on the same leg as the popliteal artery blood flow measurement. A detailed description of the custom NIRS system, its validation for measuring tibial hemoglobin content, and the subsequent offline analysis can be found in our previous work (Draghici et al., 2018). It is important to note that the NIRS technology primarily measures hemoglobin concentration in blood vessels <1mm diameter, that is, arterioles, capillaries, and venules (Boushel et al., 2001; Davis & Barstow, 2013; Grassi & Quaresima, 2016; McCully & Hamaoka, 2000). This stems from the consideration that light passing through blood vessels larger than 1 mm diameter would be completely absorbed (Mancini et al., 1994). Thus, the myogenic response assessed via the custom NIRS device reflects vasoconstriction in bone arterioles. By investigating flow in both the whole leg (via Doppler) and tibial bone (via NIRS) of the same leg, we can identify if the bone has a myogenic vasoconstriction response similar to or distinct from that in muscle.

Myogenic vasoconstriction was assessed in response to two levels of leg dependency, which increase limb perfusion pressure via hydrostatic effects of lowering the limb below heart level (Okazaki et al., 2005; Richardson & Shepherd, 1989; Stewart et al., 2003). After a 5‐min supine rest, subjects were brought to the upright position (seated with legs extended) for 5 min, followed by the dependent position (seated position with legs dependent at 90°) for 5 min.

### 2.2 Data analysis

**FIGURE 1:**
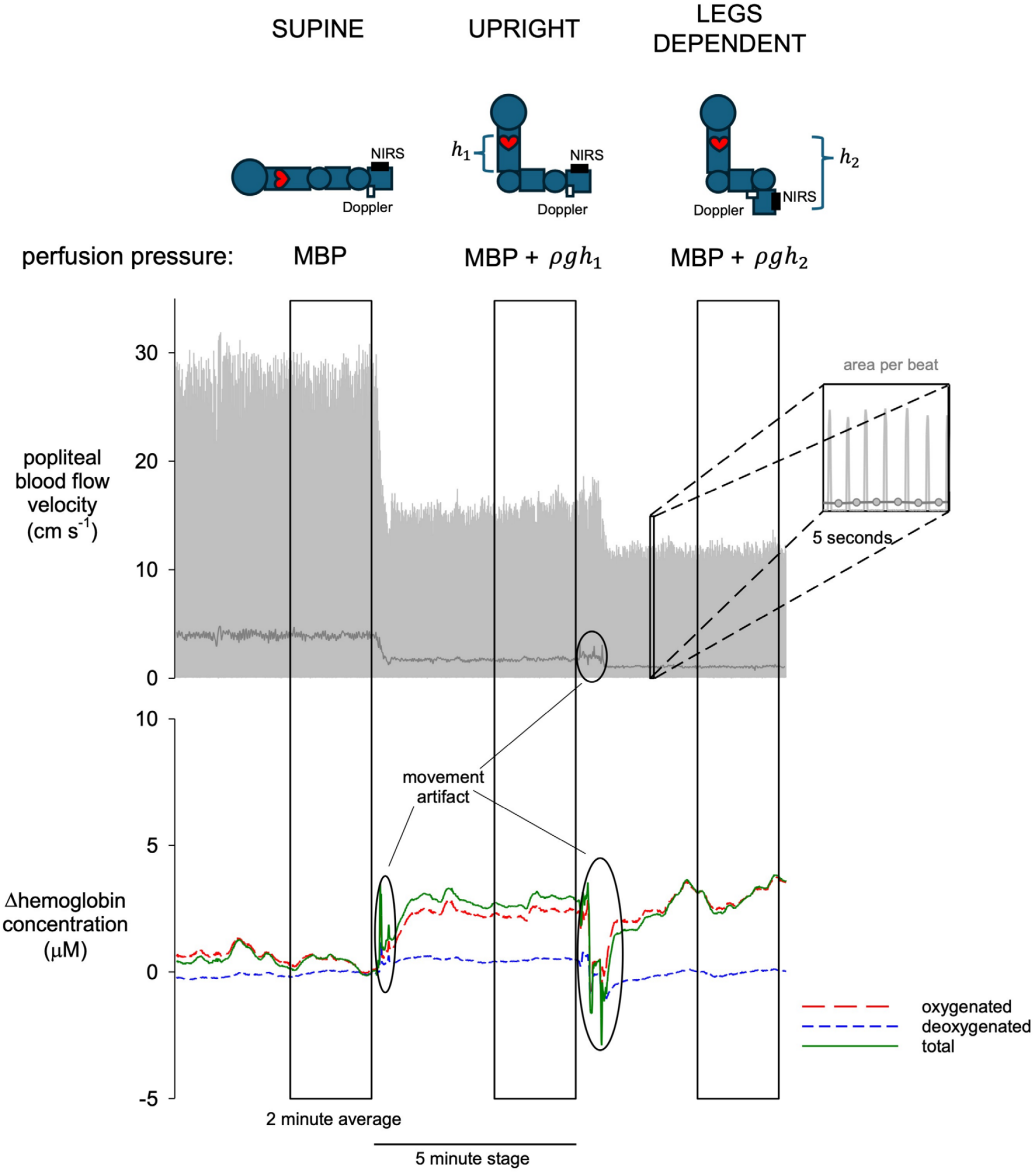
Representative blood flow response to two levels of leg dependency (supine to upright to legs dependent) assessed in the whole leg (popliteal artery blood flow velocity via Doppler ultrasound) and tibia (hemoglobin concentration via near infrared spectroscopy). Perfusion pressure was obtained as mean systemic pressure (MBP) adjusted for the hydrostatic pressure between the heart and the tibia for each position (ρ*g*h, in cm blood).

Limb perfusion pressure was calculated as mean systemic pressure adjusted for the hydrostatic pressure exerted from the heart to the tibia for each position. The hydrostatic pressure column (in mmHg) was calculated from 0.776 X distance (ρ*g*h, in cm blood) between the sternum and the NIRS location on the tibia. Mean popliteal blood flow velocity characterizing total whole leg blood flow velocity was obtained from the area of the Doppler waveform for each beat of the heart. (Figure 1) Tibial blood content (i.e., oxy‐ and deoxy‐hemoglobin) was obtained using the custom algorithm based on the extended modified Beer–Lambert law for a two‐layer model from changes in light absorption described in our prior work (Draghici et al., 2018). Total hemoglobin was obtained as the sum of oxy‐ and deoxy‐hemoglobin. The NIRS technology does not allow absolute measures of perfusion, but rather relative changes in hemoglobin content (in μM) from one state to another. Thus, changes in oxy‐ (ΔHbO_2_), deoxy‐ (ΔHHb), and total (ΔtHb) hemoglobin were obtained in the tibia during leg dependency relative to the last 60 s of supine rest. Myogenic vasoconstriction at the two levels of limb perfusion pressure (i.e., upright and dependent) was determined from the average response in ΔHbO_2_, ΔHHb, ΔtHb, and total whole leg blood flow velocity in the last 2 min of measurement to reflect stable state value.

### 2.3 Statistics

One‐sample *t*‐tests were used to determine significant changes (i.e., different from zero) in oxy‐, deoxy‐, and total hemoglobin content at each level (upright and dependent). A one‐way ANOVA with pairwise comparison was used to assess differences in heart rate, mean systemic pressure, total whole leg blood flow velocity, limb perfusion pressure, and oxy‐, deoxy‐, and total hemoglobin content from supine to each level, accounting for potential gender differences. Since the goal is physiological, not statistical, robust standard errors (SE) were used (Cleveland, 1979; Huber, 1967). Thus, all values are presented as mean ± SE; statistical significance was set at *p* ≤ 0.05. Data and statistical analyses were performed using custom software written in RStudio (R‐project, v 4.3.2).

## 3 RESULTS

**FIGURE 2:**
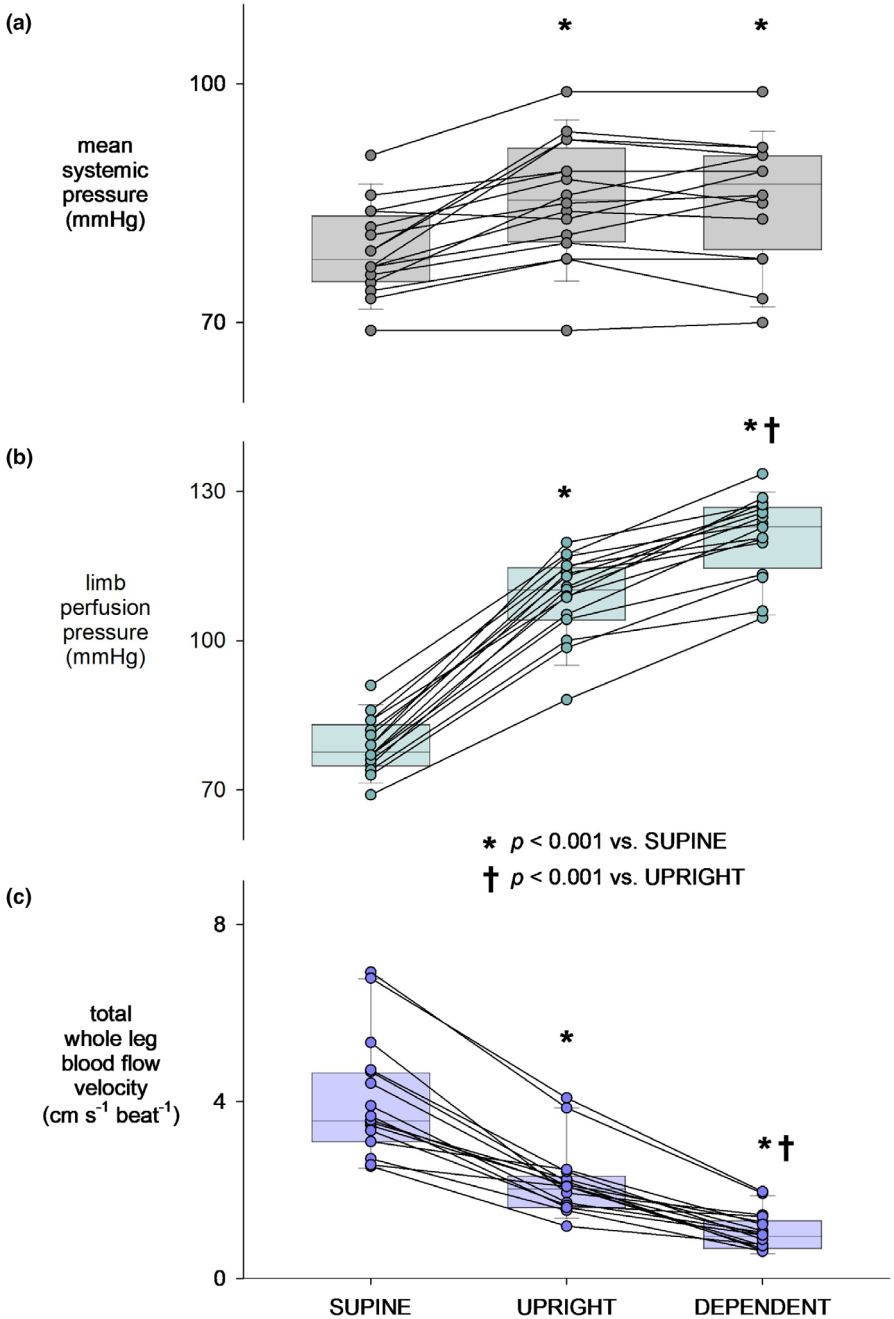
Group average and individual responses in mean systemic pressure (a), limb perfusion pressure (b), and total whole leg blood flow velocity (c) in response to two levels of leg dependency: Upright and dependent.

Group average and individual responses in mean systemic pressure, limb perfusion pressure, and total whole leg blood flow velocity are shown in Figure 2. It should be noted there were modest increases in heart rate (56 ± 2 bpm to 62 ± 2 bpm to 64 ± 3 bpm), supine to upright (*p* < 0.001 vs. supine) to leg dependent (*p* < 0.01 vs. supine; *p* = 0.04 vs. upright). Mean systemic pressure increased from supine (79 ± 1 mmHg) to upright (86 ± 2 mmHg, *p* < 0.001), without further increases in the dependent position (86 ± 2 mmHg; Figure 2a). Limb perfusion pressure increased from 79 ± 1 mmHg supine to 109 ± 2 mmHg upright, to 121 ± 2 mmHg leg dependent (both *p* < 0.001; Figure 2b). This was accompanied by decreases in total whole leg blood flow velocity from 4.1 ± 0.3 to 2.2 ± 0.3 to 1.1 ± 0.1 cm/ s^−1^ beat^−1^ (both *p* < 0.001; Figure 2c), indicating graded myogenic vasoconstriction in the whole leg.

**FIGURE 3:**
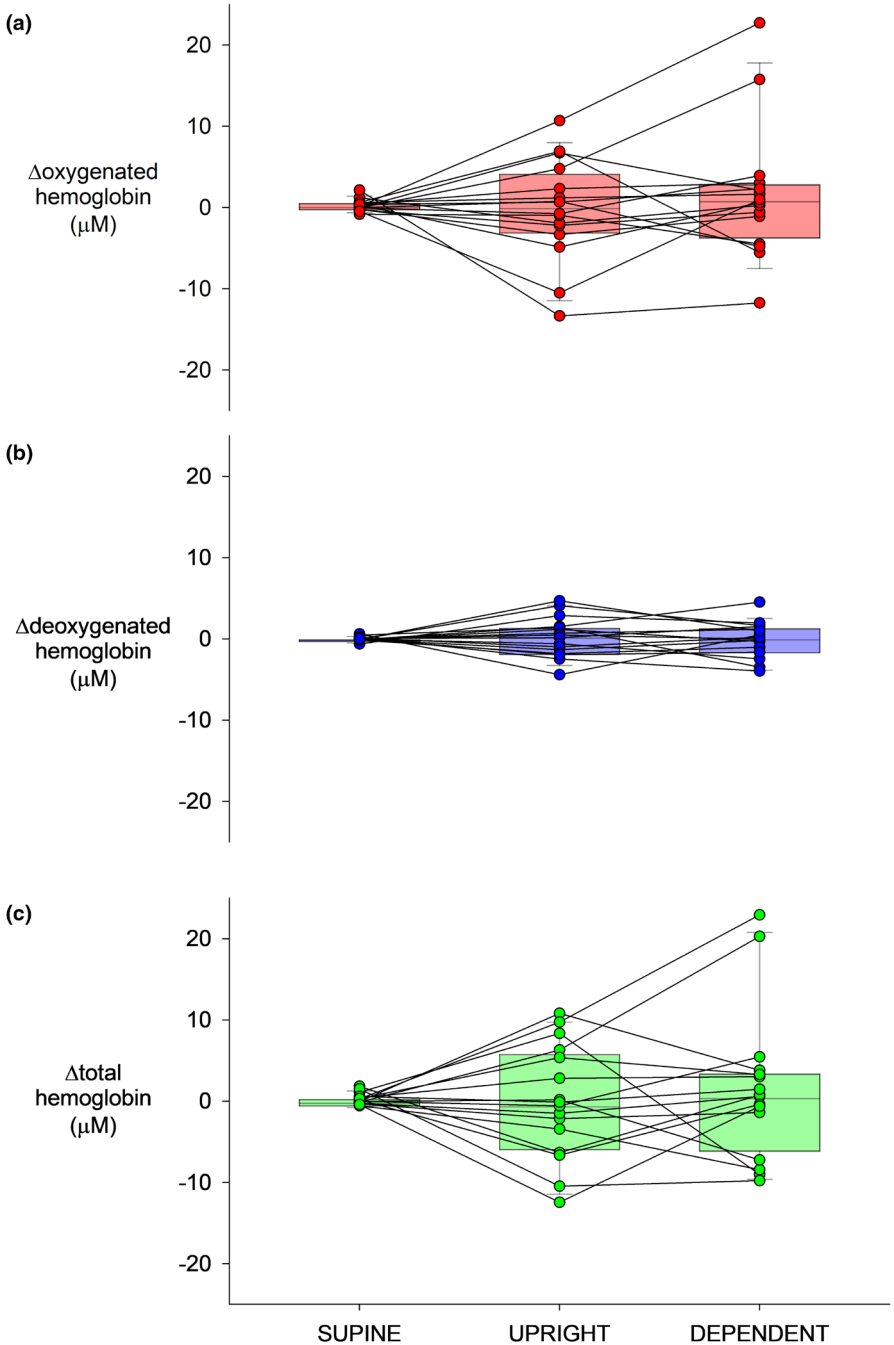
Group average and individual responses in tibial oxygenated (a), deoxygenated (b), and total hemoglobin (c) in response to two levels of leg dependency: Upright and dependent.

Group average and individual responses in tibial hemoglobin are shown in Figure 3. ΔHbO_2_ was unchanged at both pressure levels: supine 0.25 ± 0.17 μM, upright −0.24 ± 1.54 μM, and dependent 1.57 ± 2.02 μM (all *p* > 0.2; Figure 3a). Similarly, ΔHHb was unchanged at both levels: supine 0.02 ± 0.07 μM, upright 0.22 ± 0.61 μM, and dependent −0.04 ± 0.54 μM (all *p* > 0.7; Figure 3b). As a result, ΔtHb remained unchanged: supine 0.27 ± 0.17 μM, upright −0.02 ± 1.74 μM, and dependent 1.53 ± 2.30 μM (all *p* > 0.2; Figure 3c). Given the graded increases in perfusion pressure, the lack of average change in tibial hemoglobin reflects vasoconstriction. However, although all subjects had pronounced declines in whole leg blood flow velocity, (Figure 2c) tibial bone responses were more variable (Figure 3).

Although during supine rest males had higher mean systemic pressure (82 ± 2 mmHg) than females (76 ± 2 mmHg, *p* = 0.05), there were no gender differences in total whole leg blood flow velocity or tibial hemoglobin (all *p* > 0.3). In addition, there were no gender differences in heart rate, mean systemic pressure, limb perfusion pressure, and tibial hemoglobin content in upright or dependent positions.

## 4 DISCUSSION

The critical importance of the skeletal vasculature has been long recognized, but regulation of bone blood flow in humans remains poorly understood. Our prior work demonstrated that, similar to other vascular beds, sympathetic and nitric oxide mechanisms actively control bone blood flow, but with distinct differences from the whole leg (Draghici et al., 2024; Draghici & Taylor, 2021). Here, we further our previous work and provide insight into another critical regulatory mechanism— myogenic vasoconstriction. Characterizing myogenic control of blood flow to bone has been difficult in humans not only due to the inherent limitations of non‐invasive imaging of the bone vasculature, but also due to difficulties in independently assessing myogenic control without the confound of other contributing mechanisms. We used leg dependency to engage myogenic vasoconstriction in a graded fashion. The tibial vasculature evidenced an active myogenic vasoconstrictor response; however, of lesser magnitude compared to the whole leg.

In the whole leg vasculature, blood flow velocity decreased gradually with increased perfusion pressure, indicating progressive myogenic vasoconstriction. However, the tibial vasoconstrictor response was not graded and was more variable. As can be seen in Figure 3, there were individuals who evidenced no decline in tibial perfusion with either level of increased limb perfusion pressure and those who demonstrated declines at both levels. There were also those who showed responses across the two levels of increased limb perfusion that fell between this range of no evident response and robust constriction. This would suggest nonlinearities in bone myogenic responsiveness such that, within the pressure ranges we observed, there are threshold, linear, and saturation regions. By this reasoning, individuals appear to reside at different points across this relation. For example, an individual may not initially vasoconstrict at the first level of increased limb perfusion pressure because their basal myogenic tone is far below threshold, while another may not constrict further at the second level because their myogenic tone sits closer to saturation. In fact, two individuals showed increased tibial hemoglobin at both pressure levels, indicating a lack of myogenic vasoconstriction and suggesting their myogenic tone lies either below threshold or above saturation. We do not know the cause of these different responses, but they deserve further research to better characterize potential nonlinearities in bone myogenic responsiveness.

The lack of established techniques and metrics of bone blood flow means there are no available values for direct comparisons of our results, underlying one of the key motivations of this work. A few human studies employing photoplethysmography (PPG) suggested that myogenic vasodilatory responses are involved in bone blood flow regulation (Becker et al., 2018; Mateus & Hargens, 2013; Siamwala et al., 2017). However, it is unclear if the observed responses are representative of bone vasculature. The PPG and the commercial NIRS measurements use muscle‐specific properties and do not account for soft tissue contribution (skin and muscle) which is highly perfused and may cause significant artifacts, thus confounding bone measurements. Our custom NIRS system was specifically designed to incorporate properties specific to light absorption/scattering in bone and to account for the influence of skin tissue by using two detectors.

An important consideration is that when transitioning from supine to upright, the initial vasoconstriction encompasses not only local vascular mechanisms but also an increase in peripheral sympathetic outflow (Matalon & Farhi, 1979; Saito et al., 1997). However, the vasoconstrictor response observed when moving from upright to legs dependent is mediated primarily by local mechanisms rather than increased sympathetic outflow. Indeed, Snyder et al. (2012). demonstrated that myogenic vasoconstrictor responses during leg dependency remain unaffected by systemic sympathetic blockade. In addition, other factors, such as neuropeptide Y and adenosine triphosphate, may contribute to the observed vasoconstriction response. Although the design of the present study cannot exclude this possibility, previous studies employing bretylium tosylate (Crandall et al., 2002) and neural blockades (Vissing et al., 1997) found no reduction in vasoconstrictor response to increased venous pressure, suggesting that these factors are unlikely to explain our findings. Therefore, although the first postural change might engage vasoconstrictor mechanisms in addition to myogenic, the further vasoconstriction due to increased limb perfusion pressure provides clear evidence for a myogenic role in both whole leg and tibial blood flow regulation.

A limitation of the present work is that myogenic vasoconstriction was assessed only at a load‐bearing site—the tibia. It may be that myogenic vasoconstriction is more important in the legs, and so these results might not be representative of other bone vascular beds that are not load bearing, such as the radius, ulna, or humerus. However, our current NIRS analysis is not calibrated for assessments at other sites with different soft tissue thickness and absorption/scattering properties. Future work should define the generalizability of these responses to other sites. Additionally, an inherent limitation of the NIRS technology is that it provides only relative changes in perfusion from one state to another and cannot be used for an absolute measure of perfusion. However, as blood content increases, there is a proportional increase in NIRS signal (and vice versa). Thus, the hemoglobin change detected reflects the relative change in perfusion. Moreover, given the fixed distance between the light source and the detectors, the hemoglobin content is representative of the same sample volume regardless of leg size.

Characterizing regulation of bone vasculature in young healthy adults is an essential foundation for future clinical and basic science studies to address the direct link between disease‐related vascular declines and bone loss. Understanding the underlying bone vascular physiology could pave the way for potential clinical applications, including the development of novel treatment interventions targeting the vasculature. Indeed, as we have found in our recent work in spinal cord injury (Sukhoplyasova et al., 2024), there is likely a potential direct link between bone vascular control and bone loss in numerous populations such as aging, diabetes, and anorexia.

## 5 CONCLUSIONS

The present work significantly adds to our understanding of bone blood flow regulation in humans by exploring a critical mechanism—myogenic vasoconstriction. To our knowledge, the current study is the first to directly investigate myogenic vasoconstriction in human tibial bone. Our results clearly indicate myogenic vasoconstriction plays an active role in the bone vasculature, with distinct characteristics compared to the whole leg vasculature. Furthermore, our data suggest important nonlinearities may define bone blood flow responses. This foundational understanding of bone vascular physiology in humans may open new avenues for investigating vascular mechanisms that underlie bone loss disease.

## CONFLICT OF INTEREST STATEMENT

No conflicts of interest, financial or otherwise, are declared by the authors.

## ETHICS STATEMENT

The study reports outcomes from a trial registered as “Exploration of Blood Flow Regulation to Bone in Humans” (ClinicalTrials.gov Identifier NCT04083794). This study was approved by the Institutional Review Board at Partners Healthcare (2018P003133). The study conformed with the standards set by the Declaration of Helsinki and subsequent revisions, and all participants provided written informed consent prior to commencement of the study.

## Data Availability

The data underlying this article will be shared upon reasonable request to the corresponding author.
